# Association of uric acid levels with cardiac syndrome X: A meta-analysis

**DOI:** 10.3389/fphys.2022.976190

**Published:** 2022-10-03

**Authors:** Wu Zu, Chen-Chen Li, Xin-Yu Wang, Qiu-Shi Li, Bing Liu

**Affiliations:** ^1^ Department of Cardiology, General Hospital of Fuxin Mining Industry Group of Liaoning Health Industry Group, Fuxin, China; ^2^ Department of Nephrology, General Hospital of Fuxin Mining Industry Group of Liaoning Health Industry Group, Fuxin, China; ^3^ Department of Emergency, General Hospital of Fuxin Mining Industry Group of Liaoning Health Industry Group, Fuxin, China

**Keywords:** uric acid levels, cardiac syndrome X, endothelial dysfunction, system review, meta-analysis

## Abstract

**Objective:** The pathogenesis of elevated uric acid (UA) levels in patients with cardiac syndrome X (CSX) is unclear, and the results presented in recent papers on UA levels in patients with CSX are controversial. Therefore, we conducted a meta- analysis to assess the relationship between UA levels and CSX.

**Methods:** Three databases, including the Web of Science, EMBASE and PubMed, were systematically searched until January 2022. Fixed-effect and random-effects models were used to analyze the relationship between UA levels and CSX. Subgroup analysis and sensitivity analysis were also performed.

**Results:** Six studies involving 406 CSX patients and 267 non-CSX were included. Our results showed a significant relationship between UA levels and CSX, with a pooled SMD of 0.68 (95% CI 0.37 to 1.00; *p* < 0.0001). We also found a close relationship between UA levels and CSX for patients ≥ 55 years old (SMD:0.70, 95% CI: 0.41 to 0.99, *p* < 0.00001), for patients < 55 years old (SMD: 0.68, 95% CI: 0.25 to 1.12, *p* =0 .002), for women ≥ 60% (SMD: 0.77, 95% CI: 0.33 to 1.14, *p* =0 .0004), for women < 60% (SMD: 0.61, 95% CI:0.23 to 0.98, *p* =0 .001), for BMI ≥ 28 Kg/m^2^ (SMD :0.61, 95% CI: 0.23 to 0.98, *p* =0 .001), for BMI < 28 Kg/m^2^ (SMD:0.75, 95% CI: 0.31 to 1.19, *p* =0 .0009), for publication years ≥ 2012 (SMD :0.69, 95% CI: 0.23 to 1.15, *p* = 0.003), for publication years < 2012 (SMD:0.73, 95% CI:0.41 to 1.05, *p* < 0.00001), and for Turkey (SMD:0.75, 95% CI:0.38 to 1.11, *p* <.0001). Sensitivity analysis showed that the pooled results remained consistent after removing any one study or converting the random-effects model to fixed-effects model.

**Conclusion:** Our results indicated a strong association between high UA levels and CSX. However, more well-designed studies are needed to investigate whether early treatment of hyperuricemia can reduce the incidence of CSX.

## 1 Introduction

Cardiac syndrome X (CSX), also known as microvascular angina (MVA), usually presents with angina pectoris or angina-like symptoms, ST-segment depression on electrocardiogram (ECG) or exercise treadmill test, however with completely normal coronary angiography (Crea and Lanza, 2004; Kaski, 2004). Patients with CSX account for 10–30% of all patients undergoing coronary angiography for chest pain. The pathogenesis of this disease is not clear, however it may be related to endothelial dysfunction (Hurst et al., 2006).

Uric acid (UA) is the final product of purine metabolism. UA reduces the synthesis of nitric oxide (NO) by taking advantage of the oxidation of the accompanying superoxide and inhibiting the action of NO synthase. This leads to impaired vascular endothelial relaxation (Jadhav et al., 2006). High UA levels has become a serious public health problem. A study, conducted in 2017, showed that the prevalence of hyperuricemia was 13.0% (18.5% in men and 8.0% in women) (Wu et al., 2017). High UA levels is an independent risk factor for several cardiovascular and metabolic diseases, including chronic kidney disease, hypertension, diabetes and atherosclerosis (Bonakdaran and Kharaqani, 2014; Borghi et al., 2015; Johnson et al., 2018). Sakr et al. (2009). reported that UA contributes to endothelial dysfunction, which may not be related to CSX. Acikgoz et al. (2012) found that serum UA levels was an independent predictive risk factor for CSX. At present, there is no meta-analysis to study the relationship between UA levels and CSX. Therefore, in order to further evaluate the relationship, we conducted meta-analysis.

## 2 Methods

### 2.1 Search strategy

Standard guidelines for meta-analysis (PRISMA) were applied (Stewart et al., 2015). Studies involving CSX and plasma or serum UA levels (mean ± SD) were included. In order to find relevant original articles, we conducted a comprehensive search in the database, including the Web of Science, EMBASE and PubMed, using the following terms: “uric acid,” “UA,” “microvascular angina,” “Cardiac syndrome X,” “MVA,” and “CSX.”

### 2.2 Inclusion and exclusion standard

Inclusion criteria were as follows: All participants were over 18 years old; studies were limited to humans; studies featured raw data and were published in English. The diagnosis of CSX was based on one of the following three conditions 1) typical angina pectoris, ST segment depression during exercise load test and normal coronary angiography; 2) index of microcirculatory resistance ≥ 25; 3) coronary flow reserve ≤ 2.5. The control group displayed no CSX. There was no significant difference between the control group and the CSX group with respect to age and body mass index (BMI).

Exclusion criteria were as follows: Patients with coronary heart disease, acute coronary syndrome, left ventricular hypertrophy, atrial fibrillation, congenital heart disease, renal and hepatic failure, acute and chronic inflammatory diseases, excessive alcohol consumption and history of diuretic use. Also not included were duplicate studies, abstracts, letters, reviews and case reports.

### 2.3 Data extraction

Information extracted from each study included the first author’s name, publication year, country, study design, sample, sample size, age, sex, BMI, and UA levels (mean ± SD) for both the CSX group and the control group.

### 2.4 Statistical analysis

We used the Newcastle Ottawa scale (NOS) to assess the quality of each study (Stang, 2010). Meta-analysis was conducted by review manager 5.3. Heterogeneity was assessed by calculating the *I* ^2^ index. *I* ^2^ values < 25%, 25–50%, 50–75% and 75–100% were considered to be homogeneous, low, medium and high heterogeneity levels, respectively.When the *I* ^2^ value was > 50%, the random effects model (REM) was applied. Otherwise, the fixed effects model (FEM) was used. The pooled standard mean difference (SMD) of different studies and corresponding 95% confidence intervals (CIs) were used to estimate the link between UA levels and CSX. Sensitivity analysis was repeated and the impact of each study in the meta-analysis was evaluated by deleting different individual studies each time or converting the random-effects model to fixed-effects model.

## 3 Results

### 3.1 Study processing

**FIGURE 1:**
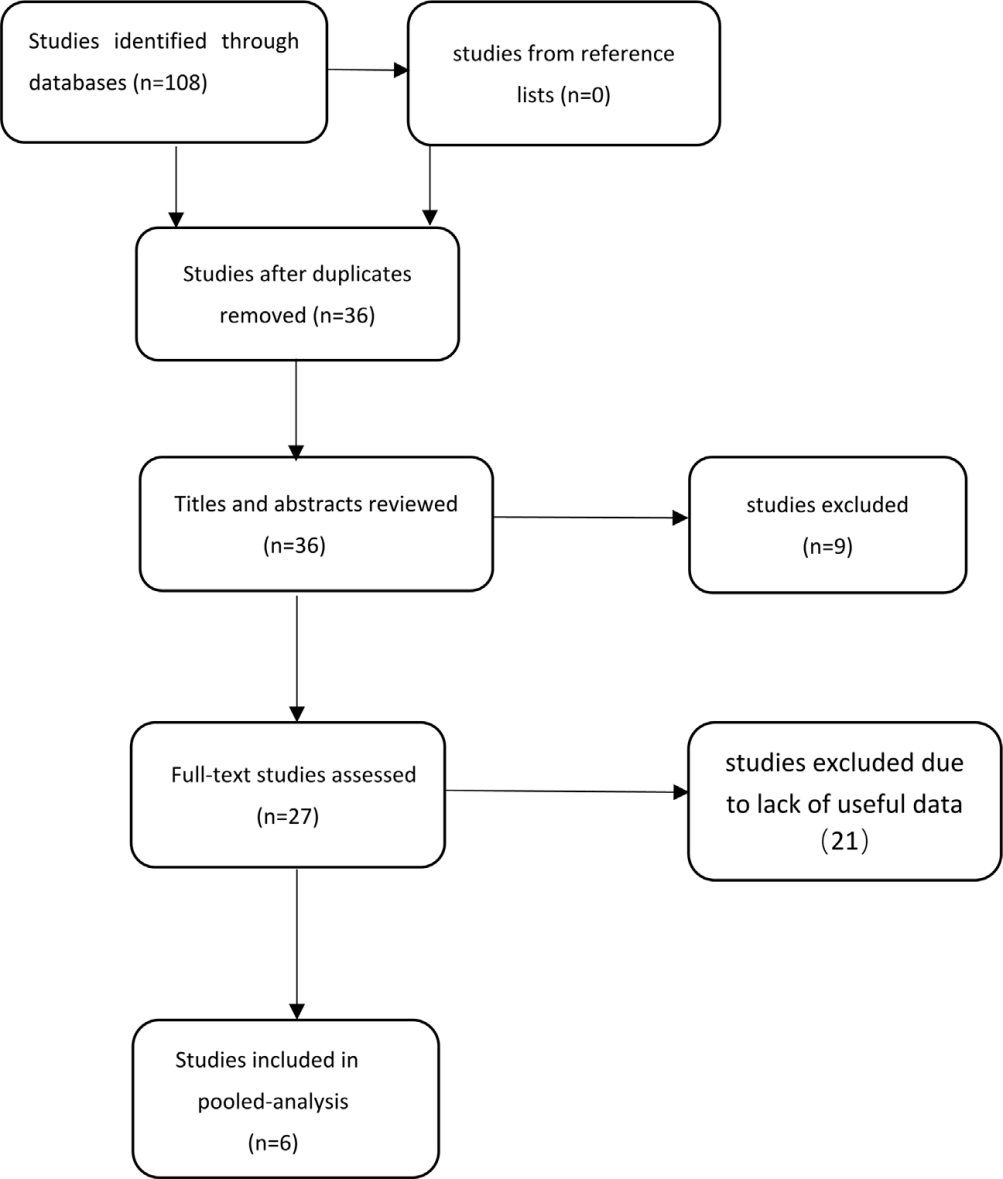
Steps of document retrieval.

First, 108 potentially relevant studies were found from our database and 0 from reference lists using our search strategy. Secondly, 36 studies were retained after excluding duplicate studies. After screening titles and abstracts, nine studies that did not meet the inclusion and exclusion criteria were excluded. Third, after scanning the entire body of the 27 retained studies, 21 studies were excluded due to lack of useful data. Finally, six studies were included in the meta-analysis (Eroglu et al., 2009; Sakr et al., 2009; Acikgoz et al., 2012; Elbasan et al., 2013; Bozcali et al., 2014; Saito et al., 2020). The steps for document retrieval are shown in Figure 1.

Pooled analysis involved six studies, a total of 406 CSX patients and 267 non-CSX patients, from case-control studies. All studies were published between 2009 and 2020. One study ^14^reported results separately for patients with normal coronary flow (group A) and patients with slow coronary flow (group B). Another study (Eroglu et al., 2009) reported women (group A) and men (group B). A study (Sakr et al., 2009) reported the population of Egypt, another study (Saito et al., 2020) reported the population of Japan, and four studies reported the population of Turkey (Eroglu et al., 2009; Acikgoz et al., 2012; Elbasan et al., 2013; Bozcali et al., 2014). Tables 1, 2 list the characteristics of the included studies.

**TABLE 1:** Description of included studies.

Study	Year	Country	Design	Sample	Sample size (CSX/CG)	NOS
Sakr	2009	Egypt	Case-control	Serum	21/10	7
Eroglu	2009	Turkey	Case-control	Serum	100/50	6
Acikgoz	2012	Turkey	Case-control	Serum	50/40	5
Elbasan	2013	Turkey	Case-control	Serum	113/41	7
Bozcali	2014	Turkey	Case-control	Serum	115/74	8
Saito	2020	Japan	Case-control	Serum	7/11	5

CSX, cardiac syndrome X; CG, control group; NOS, Newcastle Ottawa scale.

**TABLE 2:** Participant characteristics.

	Mean (SD) age, y		Women, sex, n (%)		Mean (SD)BMI,Kg/m^2^	
	CSX	CG	CSX	CG	CSX	CG
Sakr	49.7 (7.3)	46.8 (6.8)	11 (52)	5 (50)	31.6 (2.7)	31.1 (4.9)
Eroglu	52.0 (6.6)	50.1 (8.9)	64 (64)	32 (64)	27.1 (9.1)	27.0 (7.0)
Acikgoz	51.0 (1.9)	53.0 (10.2)	28 (56)	27 (68)	28.0 (4.0)	27.1 (4.5)
Elbasan	52.5 (7.1)	54.2 (6.0)	72 (64)	23 (56)	27.1 (2.5)	27.3 (2.3)
Bozcali	55.43 (8.71)	54.53 (10.07)	79 (69)	43 (58)	25.81 (2.47)	25.93 (2.08)
Saito	63.9 ± 15.2	67.3 ± 13.0	6 (86)	7 (64)	NG	NG

CSX, cardiac syndrome X; CG, control group; BMI, body mass index; SD, standard deviation; NG, not given.

### 3.2 Meta-analysis

**FIGURE 2:**
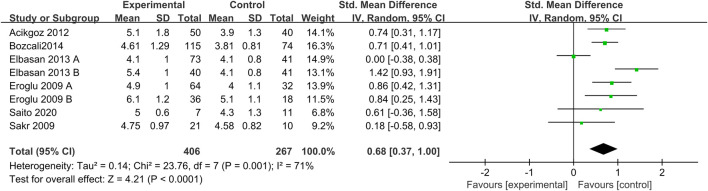
Forest plot for the connection between CSX and UA levels.

The forest map results of the relationship between UA levels and CSX are shown in Figure 2. Our results showed a significant relationship between UA levels and CSX, with a pooled SMD of 0.68 (95% CI 0.37 to1.00; *p* < 0.0001). Due to moderate heterogeneity, REM was used to calculate the pooled data.

### 3.3 Subgroup analysis

#### 3.3.1 Age

Age ≥ 55 years old was significantly associated with a SMD of 0.70 (95% CI 0.41 to 0.99, *p* < 0.00001) based on the FEM. Meanwhile, age < 55 years old was significant with a SMD of 0.68 (95% CI 0.25 to 1.12, *p* =0 .002) (Table 3).

**TABLE 3:** Results of subgroup analysis among CSX vs. CG.

Subgroup	Studies included(N)	Sample Size (CSX/CG)	Chi Square (*df*)	*p* value	Pooled SMD(95%)	Heteroge-neity (*I* ^*2*^)
Age ≥ 55 (years)	2	122/85	0.03 (1)	< 0.00001	0.70 [0.41–0.99]	0
Age < 55 (years)	4	284/182	23.66 (5)	0.002	0.68 [0.25–1.12]	79
Women ≥ 60%	4	335/217	21.99 (5)	0.0004	0.77 [0.33–1.14]	77
Women < 60%	2	71/50	1.63 (1)	0.001	0.61 [0.23–0.98]	39
BMI ≥ 28 Kg/m2	2	71/50	1.63 (1)	0.001	0.61 [0.23–0.98]	39
BMI < 28 Kg/m^2^	3	328/206	21.97 (4)	0.0009	0.75 [0.31–1.19]	82
Publication year ≥ 2012	4	285/207	21.00 (4)	0.003	0.69 [0.23–1.15]	81
Publication year < 2012	2	121/60	2.54 (2)	< 0.00001	0.73 [0.41–1.05]	21
Turkey	4	378/246	22.03 (5)	< 0.0001	0.75 [0.38–1.11]	77
Egypt	1	21/10	NG	0.64	0.18 [−0.58, 0.93]	NG
Japan	1	7/11	NG	0.22	0.61 [−0.36, 1.58]	NG

CSX, cardiac syndrome X; CG, control group; NG, not given; SMD, standard mean difference.

#### 3.3.2 Women

Women ≥ 60% was significant with a SMD of 0.77 (95% CI 0.33 to 1.14, *p* = 0.0004); Women < 60% was significant with a SMD of 0.61 (95% CI0.23 to 0.98, *p* =0 .001) (Table 3).

#### 3.3.3 BMI

BMI ≥ 28 Kg/m^2^ and BMI < 28 Kg/m^2^ was significant with SMD of 0.61 (95% CI 0.23 to 0.98, *p* =0 .001) and 0.75 (95% CI 0.31 to 1.19, *p* =0 .0009), respectively (Table 3).

#### 3.3.4 Publication year

Publication years ≥ 2012 and publication years < 2012 were significantly associated with SMD of 0.69 (95% CI 0.23 to 1.15, *p* = 0.0003) and 0.73 (95% CI 0.41 to 1.05, *p* < 0.00001), respectively (Table 3).

#### 3.3.5 Race

Turkey was significantly associated with SMD of 0.75 (95% CI 0.38 to 1.11, *p* < 0.0001) (Table 3).

### 3.4 Sensitivity analysis and quality assessment

Our study showed moderate heterogeneity (*I* ^*2*^ = 71%), and the results significantly affected the pooled results (*I* ^*2*^dropped from 71% to 0) after excluding Elbasan’s study (Elbasan 2013A and Elbasan 2013B). Therefore, we hypothesized that the study of Elbasan may be the source of heterogeneity in this meta-analysis. Sensitivity analysis indicated that SMD ranged from 0.82 (0.58–1.06) to 0.57 (0.29 to 0.85). The risk of publication bias was analyzed by Begg’s test (*p* > 0.05) and Egger’s regression test (*p* > 0.05). Results suggested that there was no significant publication bias in the meta-analysis.

## 4 Discussion

Our meta-analysis showed that CSX is significantly associated with UA levels and that UA levels were higher in CSX patients than controls. However, the value of *I* ^*2*^ = 71% suggested a moderate heterogeneity among studies. We performed subgroup analyses to look for sources of heterogeneity. Sensitivity analysis showed that the overall results remained unchanged when either study was excluded or after REM was converted to FEM. Therefore, we were confident in the data obtained in our study.

Previous studies have found that high UA levels are closely related to cardiovascular system diseases, including hypertension, myocardial infarction, heart failure, atrial fibrillation, arrhythmia and CSX (Eroglu et al., 2009; Acikgoz et al., 2012; Wildi et al., 2013; Bozcali et al., 2014; Ndrepepa, 2018; Kumar et al., 2020; Lanaspa et al., 2020; Watanabe and Usui, 2021)^,^. Our results also showed that increased UA levels are significantly associated with CSX. UA is involved in the development of CSX by participating in the formation of microvascular atherosclerosis. The following are the main underlying mechanisms: 1) Oxidative stress. UA is an endogenous antioxidant, which can protect against the damage caused by oxygen free radicals to the cardiovascular system. However, when patients form atherosclerotic plaques, the antioxidant capacity of UA is compromised, causing LDL and lipid peroxidation and resulting in vascular damage and accelerated plaque formation (Majidinia et al., 2016). 2) Inflammatory reaction. The production of UA is accompanied by the increase in superoxide anion free radicals, which are involved in the vascular inflammatory reaction. In addition, hyperuricemia is involved in the production of inflammatory mediators, which play an important role in atherosclerosis (Tzemos et al., 2001). 3) Vascular injury. The physical solubility of UA in blood is very low, and the precipitated urate crystals directly damage the arterial vessels. In addition, urate crystals activate platelets and increase the risk of coronary thrombosis (Russo et al., 2013; Kiyooka et al., 2014). UA is able to promote the expression of endothelin-1 gene and stimulate the proliferation of smooth muscle cells, thereby inducing endothelial cell dysfunction and aggravating CSX (Hirohata et al., 2014).

Many studies have shown that UA is an independent risk factor for cardiovascular disease in women (Sciacqua et al., 2015; Leonard and Marshall, 2018), and studies have also shown that patients with CSX are more likely to be women (Hurst et al., 2006). Consistent with previous findings, our findings demonstrate that elevated UA levels contribute to CSX in women, and the association is stronger in women ≥ 60% than in women < 60%. Age is an independent factor associated with elevated UA in women. Estradiol secreted by female ovaries can inhibit the absorption of UA and promote the excretion of UA. With increasing age, ovarian function declines and the secretion of estrogen is insufficient, which increases the levels of UA. Therefore, in adolescence, the increase of UA is not obvious, and in menopause, the levels of UA will increase significantly. Our study also confirms that elevated UA levels is associated with age in CSX, and that age ≥ 55 years is more strongly associated than age < 55 years. Oguri et al. (2017) and Fabbrini et al. (2014) found obesity was significantly associated with UA levels. Obesity leads to an increase in circulating adipocytokine levels in the body, which can lead to insulin resistance by affecting insulin’s metabolism of glucose and fat. This ultimately leads to UA generation and increased UA reabsorption in renal tubules, resulting in hyperuricemia (Ouchi, 2016). Our study found that elevated UA levels are not only associated with obesity, but also with those who are not obese. When Elbasan’s study was deleted, *I* ^2^ decreased from 71% to 0, which may be a source of heterogeneity. The reason may be that the population and study design of Elbasan’s study are different from other studies.

So far, there is no consistent conclusion regarding the association of UA levels with CSX, and our study is the first attempt to use a meta-analysis to demonstrate the mechanism behind CSX. However, our study also has limitations. First, the sample size is not enough, which may affect the accuracy of the results. Therefore, more large-scale studies are needed for confirmation. Secondly, although we explored the source of heterogeneity through subgroup analyses, we were unable to detect heterogeneity from other aspects due to insufficient data. Further, we had a limited number of studies that met our inclusion criteria. Thirdly, our results may be biased due to different measurement methods and instruments used to detect UA levels. In addition to this, because meta-analysis is often subject to publication bias, even though we found no evidence of significant publication bias in our analyses, it is worth noting that a few negative studies may have been overlooked. Finally, we cannot determine a reliable cut-off point for UA tests because we do not have raw data to draw ROC curves.

## 5 Conclusion

Our study showed that patients with CSX had elevated UA levels. In the future, more well designed studies will be needed to verify that treatment of high UA levels leads to a reduced occurrence of CSX.

## Data Availability

The original contributions presented in the study are included in the article/Supplementary Material, further inquiries can be directed to the corresponding author.
